# Immunopathogenesis of Pneumonia in COVID-19

**Published:** 2020-11

**Authors:** Shamila D. Alipoor, Hamidreza Jamaati, Payam Tabarsi, Esmaeil Mortaz

**Affiliations:** 1 Department of Molecular Medicine, Institute of Medical Biotechnology, National Institute of Genetic Engineering and Biotechnology, Tehran, Iran,; 2 Chronic Respiratory Diseases Research Center, National Research Institute of Tuberculosis and Lung Diseases (NRITLD), Shahid Beheshti University of Medical Sciences, Tehran, Iran,; 3 Clinical Tuberculosis and Epidemiology Research Center, (NRITLD), Shahid Beheshti University of Medical Sciences, Tehran, Iran,; 4 Department of Immunology, Faculty of Medicine, Shahid Beheshti University of Medical Sciences, Tehran, Iran

Coronavirus disease 2019 (COVID-19), caused by a member of the *Coronaviridae* family (SARS-CoV-2), was initially reported in Wuhan, China in late December and spread rapidly around the world, reaching a pandemic level (1). This disease has been diagnosed in almost 4.44 million people globally, resulting in more than 302,000 deaths until 15 May, 2020 (2). In Iran, a total of 115,000 confirmed cases of COVID-19 were reported until May 2020, with a fatality rate of 5.118%.

Coronaviruses are enveloped positive-stranded RNA viruses with an approximate size of 80–120 nm. They contain the longest viral RNA genomes of all RNA virus families (3). The whole genome sequence of this novel virus contains 29,903 nucleotides. SARS-CoV-2 is closely related to bat-derived SARS-like coronaviruses, sharing 79% nucleotide identity with SARS-CoV (4, 5). Also, viral capsid proteins, including spike glycoprotein (S protein), play an important role in the cell entry and tropism in patients with COVID-19 (6). Overall, coronaviruses use a variety of receptors to enter the cells. Recent evidence shows high homology between SARS-CoV and SARS-CoV-2, as SARS-CoV-2 uses angiotensin-converting enzyme 2 (ACE2) as its receptor, similar to SARS-CoV (7).

ACE2 is an enzyme, involved in the renin–angiotensin–aldosterone system (RAAS) (8). A positive relationship has been documented between the ACE2 expression pattern and SARS-CoV-2 pathogenicity (9). Therefore, the pattern of ACE2 expression in different tissues can determine the tropism, susceptibility, symptoms, and outcomes of SARS-CoV-2 infection (10). On the other hand, targeting ACE2 may be effective in controlling the initial COVID-19 infection.

About 80% of COVID-19 cases are asymptomatic or show mild symptoms, whereas a low percentage experience severe organ failure (11). In the first stage of the disease, that is, one to two days after the infection, the virus may infect epithelial cells in the nasal cavity and initiate replication. Over the next few days, the upper and conducting airways become infected, and the virus is detectable in nasal swabs or sputum samples (12). At this time, patients show clinical manifestations, and the innate immune response is triggered. The infected epithelial cells become the major source of interferons (IFN), and CXCL10 is produced in response to IFN production. CXCL10 is an important immune factor in SARS and influenza and is considered as a disease marker in SARS (13).

COVID-19 is mostly restricted to the upper and conducting airways in about 80% of infected patients. However, in nearly 20% of patients, the disease progresses to the lung gas exchange units and results in hypoxia and ground-glass opacification (GGO) (14). The underlying cause of severe pulmonary damage may be attributed to the high expression of AEC2 in the tissue. It has been reported that type II epithelial cells in the lungs are more sensitive to infection with SARS-CoV-2 and that infected cells undergo apoptosis (14). Since these cells are the main contributors to surfactant secretion, the reduced surfactant level in the alveoli upon viral destruction of pneumocytes causes the alveoli to collapse, which subsequently leads to pneumonia and acute respiratory distress syndrome (ARDS) in severe cases (15).

The pathological pulmonary damage caused by COVID-19 may be directly related to the viral destruction of alveolar and bronchial epithelial cells or mass production of pro-inflammatory cytokines (cytokine storm) (16). Nonetheless, massive alveolar damage and progressive respiratory failure are the leading causes of mortality in critically ill patients. Although the viral load of nasopharyngeal specimens decreases 10–15 days after the onset of symptoms in COVID-19 patients, pathological alveolar damages continue to worsen (17). It is worth mentioning that the viral titer of nasopharyngeal specimens may not be a proper marker of the viral titer in tissues, as autopsies have shown high concentrations of the virus in the lungs (18, 19). However, a recent study found no viral cytopathic changes in the lung samples of five severe COVID-19 patients (20).

Regarding the impact of cytokine storm on pulmonary damage due to SARS-COV-2 infection, it has been shown that defects in the early control of viral infection due to delayed type I IFN induction leads to the influx of hyperinflammatory neutrophils, monocytes, or macrophages to the lungs, as well as the mass production of proinflammatory cytokines (21). In COVID-19, the cytokine storm may result in an uncontrolled systemic inflammatory response, ARDS, multiple organ failure, and death in severe cases (21). Also, the level of proinflammatory cytokines, such as interleukin 6 (IL-6), IL-2, IL-10, and tumor necrosis factor alpha (TNF-α), highly increases in the serum of severe COVID-19 cases (22). The increased total count of neutrophils, besides the reduced total lymphocyte count, seems to be associated with disease severity and death (12). Moreover, the increased level of IL-6 is associated with T-cell exhaustion and disease severity (23, 24).

The immunohistochemistry (IHC) findings have indicated the presence of immune cells, including CD3, CD4, CD8, CD20, CD79a, CD5, CD38, and CD68, in the lung tissues of patients (25). The tissue repair process and aberrant wound healing lead to severe scarring and massive pulmonary interstitial fibrosis in the lung tissues (26). Also, in patients with COVID-19-induced endotheliitis, damage to endothelial lung cells leads to abnormalities in blood coagulation, fibrinolysis, and disseminated intravascular coagulation (DIC), which lead to the progression of pneumonia and induce a systemic microcirculatory dysfunction in the lungs (27).

Computed tomography (CT) scans indicate the rapid progression of pneumonia in the lungs during the viral infection process. Based on CT findings, all patients show bilateral pneumonia with GGO and initial consolidations. More prominent consolidations appear over time and worsen over the last few days before death, especially in radiographic images (28). Pulmonary consolidation with proliferated fibroblast; extracellular matrix and fibrin forming clusters in air space and in some cases intra-alveolar neutrophilic infiltration and superimposed bacterial bronchopneumonia were indicated in the most of radiography images of patients (29).

Differentiation of COVID-19 from other types of bacterial pneumonia on CT images is difficult, as CT images largely overlap (30). The main CT feature of COVID-19 pneumonia is the presence of GGO; however, the distribution of GGO in COVID-19 patients is different from pneumonia with a bacterial origin (31). In COVID-19 cases, GGO typically has a peripheral and subpleural distribution, with the involvement of multiple lobes, particularly the lower lobes (31). Also, the features of COVID-19 pneumonia are distinctive due to the absence of centrilobular nodules and mucoid impactions (31).

The pulmonary IHC examination of five patients with severe COVID-19 showed that pneumonitis is due to the complement-associated microvascular injury and progressive thrombosis (20). It was found that inflammatory reactions to the cytokine storm trigger endothelial activation and induce alveolar damage and endotheliitis, as well as progressive microvascular pulmonary thrombosis in the lungs, leading to impaired microcirculation (32). This process may also involve the microvascular bed of other organs, causing multiple organ failure and death (33). However, there is currently a lack of pathological data on COVID-19 pneumonia, based on autopsy or biopsy results. The pathological features of COVID-19 greatly resemble those of SARS and Middle East respiratory syndrome (MERS) (28).

Overall, the main histological findings of the lungs represent patchy necrosis, hyaline membrane formation, and hyperplasia of type II pneumocystis, which are associated with diffuse alveolar damage and injury to gas-exchange surfaces (12). The whole lung tissue has a diffuse congestive appearance or shows partly hemorrhagic necrosis on gross examination. Also, several multinucleated giant cells and intracytoplasmic viral inclusion bodies have been detected. Moreover, in two lung cancer patients, who were in the early phase of COVID-19 pneumonia, histological examinations revealed edema, focal reactive hyperplasia of pneumocystis with patchy inflammatory cell infiltration, and multinucleated giant cells (34).

In conclusion, although management of COVID-19 patients is important for reducing the mortality rates, to overcome this pandemic, it is crucial to deeply understand the mechanisms of this disease. It seems that a pathological perspective toward this disease can be helpful in understanding the underlying mechanisms and changes in the respiratory tract. However, pathological evidence of COVID-19 biopsies is generally poor, and major efforts are needed to expand our knowledge in this area.
